# Complex inheritance in Pulmonary Arterial Hypertension patients with several mutations

**DOI:** 10.1038/srep33570

**Published:** 2016-09-15

**Authors:** Guillermo Pousada, Adolfo Baloira, Diana Valverde

**Affiliations:** 1 Dept. Biochemistry, Genetics and Immunology, Faculty of Biology, University of Vigo, As Lagoas Marcosende S/N, 36310 Vigo, Spain; 2 Instituto de Investigación Sanitaria Galicia Sur, (IIS-Galicia Sur), Vigo, Spain; 3 Complexo Hospitalario Universitario de Pontevedra, Servicio de Neumología, Pontevedra, Spain

## Abstract

Pulmonary Arterial Hypertension (PAH) is a rare and progressive disease with low incidence and prevalence, and elevated mortality. PAH is characterized by increased mean pulmonary artery pressure. The aim of this study was to analyse patients with combined mutations in *BMPR2*, *ACVRL1*, *ENG* and *KCNA5* genes and to establish a genotype-phenotype correlation. Major genes were analysed by polymerase chain reaction (PCR) and direct sequencing. Genotype-phenotype correlation was performed. Fifty-seven (28 idiopathic PAH, 29 associated PAH group I) were included. Several mutations in different genes, classified as pathogenic by *in silico* analysis, were present in 26% of PAH patients. The most commonly involved gene was *BMPR2* (12 patients) followed by *ENG* gene (9 patients). *ACVRL1* and *KCNA5* genes showed very low incidence of mutations (5 and 1 patients, respectively). Genotype-phenotype correlation showed statistically significant differences for gender (p = 0.045), age at diagnosis (p = 0.035), pulmonary vascular resistance (p = 0.030), cardiac index (p = 0.035) and absence of response to treatment (p = 0.011). PAH is consequence of a heterogeneous constellation of genetic arrangements. Patients with several pathogenic mutations seem to display a more severe phenotype.

Pulmonary Arterial Hypertension (PAH; OMIM #178600, ORPHA 422) is a progressive, poorly characterized disease with low incidence and prevalence in the general population^1^, and a poor prognosis in terms of quality of life, morbidity and mortality^2^. Pulmonary circulation in PAH is characterized by an increased mean pulmonary arterial pressure at rest ≥25 mmHg^2^,^3^. The aetiology is quite diverse, resulting in a large variability at both clinical and genetic levels^4^ which further complicates the patient management, a systematic diagnostic evaluation and finally, lead to a premature heart failure and death^5^.

In the V World Symposium on Pulmonary Hypertension in Nice^3^, PAH was classified as idiopathic (IPAH) when the origin is unknown, heritable (HPAH) when the disease is inherited within an autosomal dominant pattern with incomplete penetrance, or associated when other conditions co-occur (APAH)^3^. The global incidence is 2–5 cases per million per year^4^. However, in countries as USA or France this value is lower, 1–2 and 2.4 cases per million per year, respectively^6^,^7^, whereas Scotland shows a higher incidence with 7.6 cases per million per year^8^. Finally, in Spain this value is 3.3 cases per million per year^1^,^9^. There is a female predominance in patients with PAH, with a gender ratio of 1.7:1 female to male^10^,^11^, as it has been widely reported.

The bone morphogenetic protein type 2 receptor gene (*BMPR2;* MIM #600799), a member of the transforming growth factor (TGF-β) superfamily, was the first causal gene identified in PAH and is mutated in approximately 10 to 40% of IPAH patients and 80% of patients with HPAH. This gene is located on chromosome 2q33^10^,^12^,^13^,^14^,^15^. Other genes have been associated with the disease, including Activin A type II receptor like kinase 1 (*ALK1/ACVRL1*; MIM #601284), located on chromosome 12q13^16^,^17^, Endoglin (*ENG;* MIM #601284)^10^, located on chromosome 9q33-34^17^,^18^, and Potassium voltage-gated channel, shakerrelated subfamily, member 5 (*KCNA5*; MIM #176267), located on chromosome 12p13^19^,^20^. Mutations in *ACVRL1, ENG* and *KCNA5* genes are less frequent than mutations in *BMPR2* gene in patients with PAH^12^,^18^. Patients with a pathogenic mutation in these genes develop a more severe phenotype and have an earlier age at diagnosis^12^,^13^,^18^. Recently, new genes related to PAH have been described as Potassium Channel Subfamily K, Member 3 (*KCNK3;* MIM #603220)^21^, Caveolin-1 (*CAV1;* MIM #601047)^22^, Cerebellin 2 Precursor (*CBLN2*; MIM #600433)^23^ or T-box 4 (*TBX4*; MIM #601719)^24^. Besides, there are genetic modifiers that affect PAH pathogenicity in combination with mutations in those genes already described^25^,^26^,^27^.

Recent findings point out that the penetrance and expressivity of PAH are likely to be directed by the mutational load of all genes involved in the disease. Thus, we aimed to analyse here the implication of harbouring a range of pathogenic mutations in PAH. In addition, we tried to establish a genotype-phenotype correlation between clinical and hemodynamic features of patients with several pathogenic mutations.

## Results

### Description of the cohort

**Figure 1:**
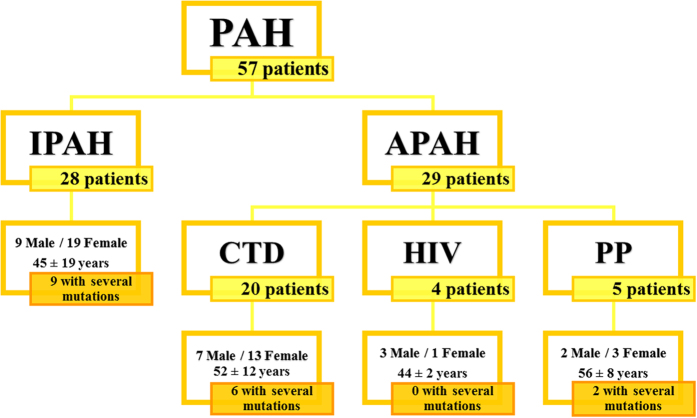
Graphical representation of the patients included in this study and their clinical features. Age displayed is the age at diagnosis. PAH: Pulmonary Arterial Hypertension; IPAH: Idiopathic Pulmonary Arterial Hypertension; APAH: Associated Pulmonary Arterial Hypertension; CTD: connective tissue disease; HIV: Human Immunodeficiency virus; P-P: Porto-pulmonary hypertension.

Fifty-seven unrelated, Caucasian PAH patients (28 idiopathic, 20 associated to connective tissue disease, 4 related to HIV and 5 porto-pulmonary hypertension) (Fig. 1) were included. At the time of diagnosis 8 patients were in functional class (FC) I, 20 patients in FC II, 25 patients in FC III and 4 in FC IV (Table 1). This cohort has been partially characterized in previous studies^12^,^25^,^28^. We have recruited patients during the last years and we performed several genetic analyses with them. The clinical description is so similar for the cohort, but for the genotype-phenotype correlation, we select only those patients of interest.

### Mutational study of *BMPR2, ACVRL1, ENG* and *KCNA5* genes

**Figure 2:**
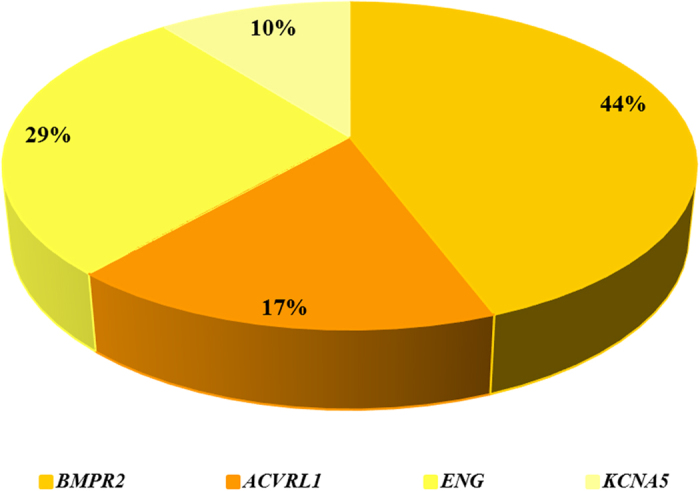
Graphical representation of the four genes analyzed here for the 57 patients included. The gene with more implication in these patients is *BMPR2*, followed by *ENG* gene and, finally, *ACVRL1* and *KCNA5* genes.

After mutational screening of *BMPR2*, *ACVRL1*, *ENG* and *KCNA5* genes, we identified pathogenic mutations in 72% (40) patients. *BMPR2* was the gene with a greater number of pathogenic mutations (44% of patients with mutations), followed by *ENG* (29%), *ACVRL1* (17%) and, finally *KCNA5* (10%) gene (Fig. 2). These results have been partially reported in Pousada *et al.*^12^ and Pousada *et al.*^28^.

**Figure 3:**
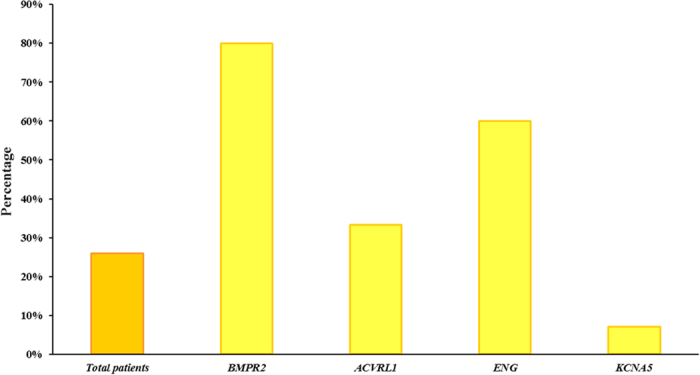
Contribution of analyzed genes in patients with several pathogenic mutations. Patients with several mutations are 26% of total and *BMPR2* genes is mutated in a large number of patients.

During the mutational analysis, we found a high percentage of patients, 26% (15 patients), with several mutations classified as pathogenic after *in silico* analysis. Among them, some patients had several mutations in the same gene whereas others harboured several mutations in different genes (Table 2). Besides, 12 of these patients were carriers of at least one mutation in *BMPR2* gene. *ENG* gene was the second most important gene involved, with 9 patients showing a mutation in this gene. However, *ACVRL1* and *KCNA5* genes were less represented, since they were mutated only in 5 and 1 patients, respectively (Fig. 3). None of these mutations were detected in 55 control samples. The variants were analyzed with an exhaustive *in silico* analysis with bioinformatics tools (Tables 3 and 4).

**Figure 4:**
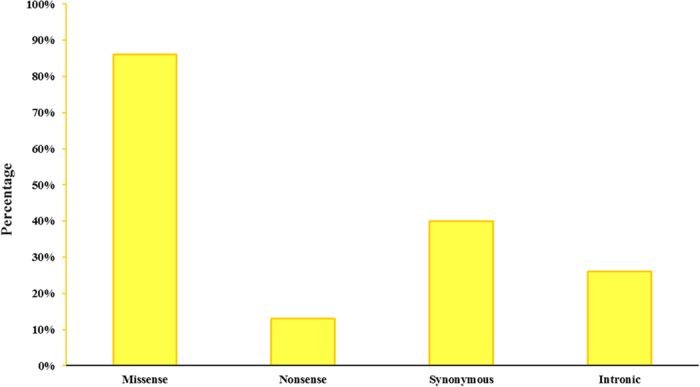
Graphical representation of pathogenic mutations type found in patients with more than one pathogenic mutation. Missense mutations are the most frequent in our patients, unlike nonsense mutations.

Focusing on patients with several mutations, all the mutations identified here were located in coding region for *BMPR2*, *ACVRL1* and *KCNA5* genes, and also in intronic junctions for *ENG* gene. Moreover, missense changes accounted for 86% of total, whereas nonsense mutations were only identified in 13% of patients. Synonymous changes and intronic variants were detected in 40% and 26% of these patients, respectively. These results are shown in Fig. 4.

We found several mutations in *BMPR2* gene in a 20% of patients with several mutations included in this study. In addition, we detected only one patient that showed two pathogenic mutations in *ENG* gene.

### Genotype correlation with clinical and hemodynamic parameters

Clinical and hemodynamic parameters were compared between patients with several mutations and patients with only one pathogenic mutation. We also performed genotype-phenotype correlation between patients with several pathogenic mutations and patients without mutations. The statistical variables considered here were gender, age at diagnosis, mean pulmonary arterial pressure (mPaP), systolic pulmonary arterial pressure (sPaP), pulmonary vascular resistance (PVR), cardiac index (CI), 6 minute walking text (6MWT), PAH type (IPAH vs APAH) and response to treatment. Patients who did not respond were treated with Phosphodiesterase 5 Inhibitors. Variables were categorized according to the best cut off point by ROC curve.

Regarding to the correlation between patients with several mutations and patients with a single mutation, we found statistically significant differences for gender (p = 0.045), with a greater number of women with several mutations, the age at diagnosis (p = 0.035), showing disease symptoms 11 years earlier, and a significantly higher PVR (p = 0.030) than patients with single mutation. Furthermore, patients with several mutations showed significant differences regarding CI (p = 0.035) and no response to therapy (p = 0.011) (Table 5). When comparing patients with several mutations and patients with no mutations, the results are quite similar to the abovementioned (Table 5). We did not find statistically significant differences according to PAH type (p = 0.401).

Three out of 57 patients in our cohort died during the mean follow up period (14 months). The first deceased patient had APAH (connective tissue disease) and he was carrier of c.251G > T (p.C84F) and c.981T > C (p.P327P) *BMPR2* mutations. The second one had IPAH and showed c.229A > T (p.I77L) and c.633A > G (p.R211R) mutations in *BMPR2* gene and c.1272 + 6A > T mutation in *ENG* gene. Finally, the last deceased patient was classified as APAH (porto-pulmonary hypertension) and harboured c.1021G > A (p.V341M) mutation in *BMPR2* gene and c.498G > A (p.Q166Q) mutation in *ENG* gene.

## Discussion

In this study, we have identified and characterized 15 out of 57 PAH patients carrying more than one pathogenic mutation in several genes related to PAH, such as *BMPR2*, *ENG*, *ACVRL1* and *KCNA5*. Twelve of these patients harboured at least one mutation in *BMPR2*, reinforcing the role of this gene in the development of PAH. On the other hand, nine patients were carriers of mutations in the *ENG* gene, representing the second gene most frequently involved in our cohort of PAH patients with several mutations. Remarkably, eight patients showed mutations in both genes. However, only five and one patients had mutations in *ACVRL1* and *KCNA5* genes, respectively. As a whole, it is difficult to elucidate the role that each of the different mutations could have had in the development of disease. Thus, the molecular pathogenic mechanism of PAH is not fully understood; in fact multiple genetic and environmental factors have been related to the disease. Many of the involved genes are part of the TGF-β signalling pathway, so several mutations in one or more genes in the same pathway could explain the reduced penetrance for PAH.

The characterization of putative missense mutations was performed by *in silico* analysis, selecting only those identified as pathogenic by at least three software tools, whereas synonymous and intronic mutations were classified as pathogenic if two bioinformatic programs that analyse splice sites gave positive results. Thus, we consider this approach is stringent enough to make an accurate classification at this level. However, it is important to note that this is only a bioinformatic prediction to characterize the nature of the change, the variants do not appear in public databases, nor detected in general population so those are pieces of evidence for the pathogenic nature of the change^29^, although functional analyses should be performed in order to identify them as clearly pathogenic.

Recently, Mallet *et al.*^30^ performed functional analysis for several *ENG* mutations. They detected 10 patients with Hereditary Hemorrhagic Telangiectasia (HHT; OMIM #187300) with more than one mutation in *ENG* or with one mutation in *ENG* and another one in *ACVLR1* gene, but after functional analysis there were not differences compared to wild-type, considering these *ENG* missense mutations as rare benign variants^30^. We detected not only missense mutations in *ENG*, but also mutations affecting the splicing process, and interestingly a high proportion of patients with *ENG* mutations harbouring an additional mutation in *BMPR2* gene. *ENG* inhibits the TGF-β pathway in endothelial cells by down-regulating the ALK5/Smad3 pathway but enhanced ALK1 signalling. As it have been described in other oligogenic diseases with a specific major gene involved in their development, mutations in other genes within the same pathway should be considered^31^. Rodríguez-Viales *et al.*^32^ published a study of two PAH families in which index patients showed one mutation in the 5′UTR region of *BMPR2* gene described by Wang *et al.*^33^ in an IPAH patient, along with another mutation in the coding region of *BMPR2* or in the *ENG* gene, respectively. They suggested that the mutation in the promoter region could explain the variable penetrance of the disease^32^ as it has been related to a decrease in the expression of *BMPR2*.

Total mutational load has been described for other pathologies, involving mutations in several genes that codify for proteins belonging to the same or related pathways, in the same individual^34^,^35^. Taking this into account, we could not discard an oligogenic inheritance model for PAH as described for others diseases, with a major gene being *BMPR2*. Approaches like next generation sequencing (NGS) analysis could give us genetic information that will help in the understanding of the molecular basis of PAH. The oligogenic inheritance might increase the risk of developing the disease and perhaps a more severe phenotype, as occurs in other diseases, like Bardet-Biedl Syndrome or Autosomal Dominant Retinitis Pigmentosa^34^,^35^,^36^.

Thirteen of our patients were carriers of a mutation in *BMPR2* gene, and four of them showed two mutations in this gene. All these changes were predicted to alter the splicing process or the conservation of the protein, producing a shorter transcript or a misfolding protein susceptible of degradation, which could prevent achieving a minimum protein translation and therefore, the development of the disease^29^,^37^. Two of these patients were carriers of a third mutation, previously described, in *ENG* gene. These mutations are located in the first exons and were predicted to affect the splicing process. Thus, mutations in *ENG* gene could prevent the correct anchoring of ENG protein in the cell membrane, impairing TGF-β/ALK1 signalling responses^33^.

On the other hand, eight patients were double heterozygotes for *BMPR2* and *ENG* mutations (one of them showed a third mutation in *ACVRL1* gene), one patient had two mutations in *ENG* gene and the remaining patients showed different combination of mutated genes: one patient was double heterozygote for *ENG* and *ACVRL1*, another two for *BMPR2* and *ACVRL1*genes and finally, one patient showed a combination of *ACVRL1* with *KCNA5* genes.

In the last years a second hit hypothesis have been proposed that two mutations, one major and other as modulator, in the same gene or different gene take place^32^,^33^. It has been described that after *BMPR2, ACVRL1* is the gene most frequently mutated in PAH patients. However, we show that *ENG* was the second gene most frequent in our cohort. All of these genes have been described to be involved in the development of the disease with or without HHT, being *BMPR2* the major causal gene and the others genetic modifiers modulating the penetrance of the disease^32^,^36^,^38^.

Although almost all mutations described in *BMPR2* gene have been established as pathogenic, others remains indeterminate, as mutations in the cytoplasmic tail that still retain capacity for downstream signalling. The pathogenic impact of others genes in the disruption of the TGF-β pathway directly or by modulating related pathways, is still unknown^39^,^40^,^41^,^42^.

None of our patients had relatives with PAH, so we could not perform segregation analysis; but none of the mutations described here were detected in 110 control chromosomes. As most of the mutations identified in PAH are private, and due to the confluence of two or more mutations in several genes, performing genotype-phenotype correlations revealed as a hard task. For this reason, the genotype-phenotype correlation has been performed grouping mutations identified on the same gene, comparing the clinical and hemodynamic parameters with patients carrying only one pathogenic mutation and also with the group of patients without pathogenic mutations.

The co-occurrence of several pathogenic mutations was more prevalent in women, which is in agreement with the higher prevalence of PAH in women^10^,^11^,^38^. However, Liu *et al.*^43^ postulated that the pathogenic mutations are more severe and prevalent in men for *BMPR2* gene, suggesting hormonal implication. Our study did not corroborate such hypothesis, but it seems that the molecular basis of this disease could be more complex in women than men. The age of diagnosis was 11 years younger in patients with several mutations as previously described by Rodríguez-Viales *et al.*^32^ and Wang *et al.*^33^. These studies reported that patients carrying one or more pathogenic mutations exhibit an early age at diagnosis than patients without mutations. PVR were also significantly higher in patients with several mutations whereas the CI was lower. Furthermore, these patients had a worse response to treatment, compared with patients with a single or none mutation. This suggests that patients with several mutations need a more specifically treatment, in some cases directed to more than one cellular pathway. Accordingly, these patients seem to have a more severe illness and a worse prognosis. These results agree with those obtained by Rodríguez-Viales *et al.*^32^, who reported patients with several pathogenic mutations with a more severe phenotype. Also, in a previous study made by our group^12^, we pointed out that patients with several pathogenic mutations may show a greater predisposition to develop the disease.

Three patients died after the follow-up period. They had an early age at diagnosis and were carriers of several pathogenic mutations. In addition, these patients did not respond to treatment, achieving a gradual increase of the characteristic phenotype of PAH leading to a premature death. These patients, as well as all cases with various pathogenic mutations, had a more severe phenotype and a higher functional class at diagnosis than patients without pathogenic mutations or with only a single one, but this small number does not allow us to perform statistical analysis.

Our results are consistent with those obtained by other authors, but the small number of patients can be considered a limitation. However, the extensive genetic study and monitoring of our patients add extra values to our results.

In summary, we report a series of IPAH and APAH patients with a high percentage of them carrying more than one pathogenic mutation in several genes. Moreover, *BMPR2* was the more frequently affected gene, followed by *ENG*, *ACVRL1* and *KCNA5* genes. Some mutations had not been previously described. We cannot rule out that patients with a single pathogenic mutation have other mutations in genes not included in this study. There is no doubt that other genes could be involved in PAH and it will be important to understand their role in the development of the disease. Patients with several pathogenic mutations seem to show a more severe phenotype. We wonder whether these additional mutations act as a second event in the development of the disease, increasing the penetrance or simply modifying the phenotype of patients.

## Material and Methods

### Patients and samples

Fifty-seven patients with idiopathic or associated PAH (group 1 of the new classification of Nice)^6^ followed in our Pulmonary Arterial Hypertension Unit were enrolled. This cohort has been described previously by our group^12^,^25^. Fifty-five healthy individuals of Spanish origin without a familial history of PAH were also included to determine their mutational frequencies, kindly provided by *Complexo Hospitalario Universitario de Vigo* (Vigo, Spain). All patients are included in the CHUVI DNA Biobank (*Biobanco del Complejo Hospitalario Universitario de Vigo*). Patients signed an informed consent and the Regional Ethics Committee approved the study (Galician Ethical Committee for Clinical Research; *Comité Autonómico de Ética da Investigación de Galicia - CAEI de Galicia*), following the clinical-ethical guidelines of the Spanish Government and the Helsinki Declaration.

Cardiac catheterization was performed using the latest consensus diagnostic criteria of the ERS-ESC (European Respiratory Society-European Society of Cardiology)^44^. PAH was considered idiopathic after exclusion of the possible causes associated with the disease. Clinical data included use of drugs, especially appetite suppressants, and screening for connective tissue diseases and hepatic disease. The study also included serology for HIV, autoimmunity, thoracic CT scan, echocardiography, right catheterization and 6 minute walking test (6MWT). Patients with PAH that could be related to chronic lung disease were excluded^12^,^25^. The criteria of good response to treatment after 6 months were: decrease of at least one functional class, increase the distance walked in the 6MWT at least 10%, no hospital admissions and no episodes of right heart failure.

### Identification of mutations in *BMPR2, ACVRL1, ENG* and *KCNA5* genes

Genomic DNA was extracted from leukocytes isolated from venous blood using the FlexiGene DNA Kit (Qiagen, Hilden, Germany) according to the manufacturer’s protocol. We used primers described by Deng *et al.*^45^ for *BMPR2* gene, by Berg *et al.*^46^ for *ACVRL1* gene, by Gallione *et al.*^47^, with minor modifications, for *ENG* gene, and by Yang *et al.*^48^ for *KCNA5* gene. Amplification of exons and intronic junctions was performed with 50 ng of genomic DNA using GoTaq® Green Master Mix (Promega Corporation, Madison, Wisconsin, USA), according to the manufacturer’s protocol. GoTaq® Green Master Mix contained MgCl_2_, dNTPs, reaction buffer and Taq DNA polymerase. PCR was performed in a GeneAmp PCR System 2700 (Applied Biosystems, Carlsbad, California, USA).

PCR products were confirmed by electrophoresis through 2% agarose gels with SYBR® Safe DNA Gel Stain (Invitrogene, San Diego, California, USA) in a Sub-Cell GT (Bio-Rad, Hercules, California, USA). HyperLadder V was used as molecular weight marker (New England Biolabs, Ipswich, Massachusetts, USA). The PCR product was purified using the Nucleic Acid and Protein Purification NucleoSpin Extract II kit (Macherey-Nagel, Düren, Germany) or ExoSAP-IT kit (USB Corporation, Cleveland, Ohio, USA). Purified PCR products were sequenced for both forward and reverse strands with BigDye Terminator version 3.1 Cycle Sequencing Kit (Applied Biosystems, Carlsbad, California, USA). The sequencing reactions were precipitated with Agencourt CleanSEQ - Dye Terminator Removal (Beckman coulter, Brea, California, USA) and analyzed in an ABI PRISM 3100 genetic analyzer (Applied Biosystems, Carlsbad, California, USA). All results were confirmed by a second independent PCR.

### Analysis of mutations

Sequence data were aligned with the reference Ensembl cDNA sequence [ENST00000374580] for *BMPR2* gene, [ENST00000388922] for *ACVRL1* gene, [ENST00000344849] for *ENG* gene and [ENST00000252321] for *KCNA5* gene, and examined for sequence variations. We use the Basic Local Alignment Search Tool (BLAST) software to align sequences and compare them with different organisms. Rare missense variants were analyzed to predict their potential pathogenicity, used combined computer algorithms: *Polyphen-2*^49^, *Pmut*^50^, Sort Intolerant from Tolerant (*SIFT*)^51^ and *MutationTaster2* software^52^. Other combined computer algorithms were used to predict whether that change could affect donor/acceptor splice sites: *HSF Human*^53^, *NetGene2*^54^, *Splice View*^54^ and *NNSplice*^54^. Fifty-five control samples were checked in order to established genetic frequencies for all mutations detected.

We classified a missense variant as a mutation when is considered pathogenic by at least three software tools. In addition, synonymous and intronic variants were classified as pathogenic if at least two out of four bioinformatics tools used to predict alterations in mRNA processing showed a new donor/acceptor splice site or if the prediction change dramatically.

### Statistical analysis

We used statistical package SPSS v19 for Microsoft. A non-parametric test (U Mann-Whitney) was used for comparisons between patients and controls, but this approach was only exploratory. To compare the different genotypes with clinical and hemodynamic variables we used the Chi-square test. Values were expressed as mean ± SD (standard deviation). P-values < 0.05 were considered statistically significant.

## Additional Information

**How to cite this article**: Pousada, G. *et al.* Complex inheritance in Pulmonary Arterial Hypertension patients with several mutations. *Sci. Rep.* **6**, 33570; doi: 10.1038/srep33570 (2016).

## Figures and Tables

**Table 1:** Clinical features and hemodynamic parameters of patients included in this study.

Clinical features and hemodynamic parameters	Total patients
*Number*	57
*Gender*	20 M/37 F
*Age at diagnosis (years*)	49 ± 16
*mPaP (mmHg*)	49 ± 14
*sPaP (mmHg*)	70 ± 19
*PVR (mmHg.l*^*−1*^*.m*^*−1*^)	8.1 ± 3.3
*CI (l.min*^*−1*^*.m*^*−2*^)	2.4 ± 0.7
*6MWT (m*)	415 ± 146
*PAH types*	28 IPAH/29 APAH
*No response to treatment*	22

Values are expressed as mean ± standard deviation; F: female, M: male; mPaP: mean pulmonary artery pressure; sPaP: systolic pulmonary artery pressure; PVR: pulmonary vascular resistence; CI: cardiac index; 6MWT: 6 minute walking test; IPAH: idiopathic pulmonary arterial hypertension; APAH: associated pulmonary arterial hypertension.

**Table 2:** Patients with several pathogenic mutations in the four genes analyzed.

Patients	Genes involved in patients with several mutations			
	*BMPR2*	*ACVRL1*	*ENG*	*KCNA5*
1 (IPAH)	c.275A > T (p.Q92L)^13^	—	c.498G > A (p.Q166Q)^29^	—
2 (IPAH)	c.190A > C (p.S64G)^13^	—	c.1272 + 6A > T^29^	—
3 (APAH)	c.251G > T (p.C84F)^13^	—	—	—
c.981T > C (p.P327P)^13^ ExAC = 0.0001675				
4 (APAH)	c.637C > A (p.R213R)^13^	—	c.498G > A (p.Q166Q)^29^	—
5 (APAH)	—	—	c.360 + 56T > A^29^	—
c.1272 + 6A > T^29^				
6 (IPAH)	c.893G > A (p.W298*)^13^	c.24A > T (p.K8N)^13^	c.1272 + 6A > T^29^	—
7 (APAH)	c.1467G > A (p.E498E)^13^	—	c.775G > A (p.V259M)^29^	—
8 (IPAH)	c.229A > T (p.I77L)^13^	—	c.1272 + 6A > T^29^	—
c.633A > G (p.R211R)^13^				
9 (APAH)	c.1021G > A (p.V341M)^13^	—	c.498G > A (p.Q166Q)^29^	—
10 (IPAH)	c.156_157delTC (p.S52Sfs*2)**	—	—	—
c.742A > G (p.R248G)**				
11 (APAH)	—	c.694T > A (p.S232T)**	c.1633G > A (p.G545S) ExAC = 0.0005205	—
12 (IPAH)	c.412C > G (p.P138A)**	c.682G > A (p.V228I)**	—	—
13 (IPAH)	—	c.682G > A (p.V228I)**	—	c.676C > A (p.P226T)**
14 (APAH)	c.742A > G (p.R248G)**	c.760G > A (p.D254N)**	—	—
15 (APAH)	c.790G > A (p.D264N)^13^	—	c.1660C > A (p.R554C)	—

IPAH: idiopathic pulmonary arterial hypertension; APAH: associated pulmonary arterial hypertension.

These mutations not where found in 1000 Genome Project and the Spanish variant server. For this reason, we don’t show the Genotype frequency values for these mutations. In ExAC database, only information for c.981T > C (p.P327P) mutation for *BMPR2* gene and c.1633G > A (p.G545S) mutation for *ENG* gene, appears.

^13^Described in ref. 12.

^29^Described in ref. 28.

^**^These mutations were described by first time in this study.

**Table 3:** Bioinformatic assessment of the pathogenic nature of missense variations.

*Mutation*	*PolyPhen-2*	*Pmut*	*Sift*	*Mutation Taster*	*NNSplice*	*NetGen2*	*Splice View*	*HSF Human*	*Score*
c.190A > C (p.S64G) *BMPR2*	Benign	Neutral	Tolerated	Disease causing	Neutral	The WT consensus sequence is not recognized	A new donor site is created	Score for donor and acceptor site decreases from 85 to 52	3
c.229A > T (p.I77L) *BMPR2*	Benign	Neutral	Damaging	Disease causing	The WT consensus sequence is not recognized	Score for the main donor site increases from 31 to 34	Neutral	A new acceptor site is created	4
c.251G > T (p.C84F) *BMPR2*	Probably Damaging	Neutral	Damaging	Disease causing	Score for the aceptor site increases from 87 to 89	Score for the main acceptor site decreases from 33 to 27	Neutral	The main donor site is not recognized	6
c.275A > T (p.Q92L) *BMPR2*	Benign	Pathologic	Damaging	Disease causing	Neutral	Score for the main acceptor site decreases from 33 to 25	Neutral	Score for donor and acceptor site increases from 59 to 89 and from 89 to 92	5
c.412C > G (p.P138A) *BMPR2*	Probably Damaging	Neutral	Damaging	Disease causing	Neutral	Neutral	Neutral	The main donor site is not recognized	4
c.742A > G (p.R248G) *BMPR2*	Benign	Neutral	Damaging	Disease causing	Neutral	Score for the main donor site decrease from 94 to 77	Neutral	Score for donor and acceptor site decrease from 87 to 57 and from 79 to 35	4
c.790G > A (p.D264N) *BMPR2*	Possibly damaging	Neutral	Damaging	Disease causing	Neutral	Score for the main donor site decreases from 94 to 92	Neutral	The main donor site is not recognized	5
c.1021G > A (p.V341M) *BMPR2*	Possibly damaging	Neutral	Damaging	Disease causing	Neutral	Neutral	The WT consensus sequence is not recognized	The main donor site is not recognized	4
c.24A > T (p.K8N) *ACVRL1*	Benign	Neutral	Tolerated	Disease causing	Score for the main acceptor site increases from 66 to 76	Neutral	Neutral	The main donor and acceptor sites are not recognized	3
c.682G > A (p.V228I) *ACVRL1*	Probably Damaging	Neutral	Damaging	Disease causing	Neutral	The main aceptor site is not recognized	Neutral	Score for main acceptor site increase from 69 to 85	5
c.694T > A (p.S232T) *ACVRL1*	Probably Damaging	Neutral	Damaging	Disease causing	Score for main donor site increase from 69 to 93	Neutral	The WT consensus sequence is not recognized	The main donor site is not recognized and a new acceptor site is created	5
c.760G > A (p.D254N) *ACVRL1*	Probably Damaging	Neutral	Damaging	Disease causing	Score for main donor site decrease from 69 to 48	Score for main donor site increase from 65 to 75	Neutral	Neutral	5
c.775G > A (p.V259M) *ENG*	Probably Damaging	Neutral	Damaging	Polymorphism	Neutral	Score for the main acceptor site increase from 35 to 37	Neutral	A new acceptor site is created	4
c.1633G > A (p.G545S) *ENG*	Probably Damaging	Pathological	Tolerated	Disease causing	Neutral	Neutral	Neutral	A new acceptor site is created	4
c.1660C > A (p.R554C) *ENG*	Probably Damaging	Pathological	Tolerated	Polymorphism	Neutral	Score for the main donor site decreases from 69 to 67	Neutral	A new acceptor site is created	4
c.676C > A (p.P226T) *KCNA5*	Probably Damaging	Pathological	Damaging	Disease causing	The WT consensus sequence is not recognized	The WT consensus sequence is not recognized	The WT consensus sequence is not recognized	The WT consensus sequence is not recognized	4

Score: number of bioinformatic tools that evidence the pathogenic nature of the variants.

**Table 4:** Bioinformatic assessment of the pathogenic nature of synonymous and intronic variations.

*Mutation*	*NNSplice*	*NetGen2*	*Splice View*	*HSF Human*	*Score*
c.633A > G (p.R211R) *BMPR2*	Neutral	Score for the main donor site increases from 92 to 94	Neutral	The main donor site is not recognized and the acceptor decrease from 89 to 56	2
c.637C > A (p.R213R) *BMPR2*	Neutral	Score for the main acceptor site decreases from 20 to 8	Neutral	Score for donor site increases from 89 to 99 and a new acceptor site is created	2
c.981T > C (p.P327P) *BMPR2*	The WT consensus sequence is not recognized	Score for the main donor site decreases from 100 to 89	Neutral	A new donor site is created	2
c.1467G > A (p.E498E) *BMPR2*	Neutral	Score for the main donor site increases from 89 to 93	The WT consensus sequence is not recognized	A new acceptor site is created	3
c.498G > A (p.Q166Q) *ENG*	Neutral	Score for the main donor site decreases from 90 to 87	A new donor site is created	Score for the main acceptor site decrease from 82 to 53	3
c.360 + 56T > A *ENG*	Neutral	Score for the main donor site decreases from 93 to 89	Neutral	A new acceptor site is created	2
c.1272 + 6A > T *ENG*	Neutral	Neutral	A new donor site is created	Score for the main acceptor site decrease from 65 to 37	2

Score: number of bioinformatic tools that evidence the pathogenic nature of the variants.

**Table 5:** Clinical and p-values for genotype-phenotype correlation comparing patients with several mutations vs patients with one pathogenic mutations.

Clinical features and hemodynamic parameters	Clinical data	Patients with several pathogenic mutations vs patients with single mutation	Patients with several pathogenic mutations vs patients without mutation
		p-value	p-value
*Number*	15	—	—
*Gender*	4 M/11 F	0.045	0.040
*Age at diagnosis (years*)	46 ± 17	0.035	0.030
*mPaP (mmHg*)	48 ± 15	0.239	0.368
*sPaP (mmHg*)	72 ± 17	0.542	0.422
*PVR (mmHg.l*^*−1*^*.m*^*−1*^)	10.9 ± 1.7	0.030	0.025
*CI (l.min*^*−1*^*.m*^*−2*^)	1.7 ± 0.5	0.035	0.018
*6MWT (m*)	389 ± 163	0.075	0.027
*PAH types*	7 IPAH/8 APAH	0.401	0.472
*No response to treatment*	10	0.011	0.125

Values are expressed as mean ± standard deviation; F: female, M: male; mPaP: mean pulmonary artery pressure; sPaP: systolic pulmonary artery pressure; PVR: pulmonary vascular resistence; CI: cardiac index; 6MWT: 6 minute walking test; IPAH: idiopathic pulmonary arterial hypertension; APAH: associated pulmonary arterial hypertension.
